# Prognostic Factors for Radiographic Progression in Patients with Seronegative Rheumatoid Arthritis

**DOI:** 10.3390/jpm11030184

**Published:** 2021-03-05

**Authors:** Eun-Jung Park, WooSeong Jeong, Jinseok Kim

**Affiliations:** 1 Division of Rheumatology, Department of Medicine, National Medical Center, Seoul 04564, Korea; ejcoke.park@gmail.com; 2 Division of Rheumatology, Department of Medicine, Jeju National University School of Medicine, Jeju 63241, Korea; dfac36@naver.com

**Keywords:** seronegative, rheumatoid arthritis, predictors, radiographic damage

## Abstract

(1) Background: It has long been suggested that seronegative rheumatoid arthritis (RA) represents a clinical entity quite distinct from that of seropositive. However, analytical studies of seronegative RA dedicated to clinical outcomes regarding radiographic progression and related risk factors are scarce. The aim of this study is to evaluate radiographic outcome and prognostic factors for radiographic progression in patients with seronegative rheumatoid arthritis. (2) Methods: Subjects included RA patients reported as seronegative for both rheumatoid factor and anti-citrullinated protein antibody, who were treated at Jeju National University Hospital in South Korea between 2003 and 2016, including follow-up of at least 2 years. All patients fulfilled 1987 ACA or 2010 ACR/EULAR RA criteria. Radiographic progression was measured by yearly change in the Sharp van der Heijde (SvdH) score during follow-up periods. Medical records, laboratory and radiographic data were retrospectively analyzed, and linear regression analysis was performed to evaluate prognostic factors for radiographic progression in patients with seronegative rheumatoid arthritis. (3) Results: In total, 116 patients with seronegative RA were observed and 43 (37.1%) patients demonstrated radiographic damage during follow-up period. Mean age at diagnosis was 48 years and 86 (74.1%) patients were female. Symptom duration at diagnosis was 1.3 years and mean follow-up duration was 5.2 years. Patients with radiographic damage at diagnosis were 14 (12.1%) and mean SvdH score was 6.8 at diagnosis. Radiographic damage and SvdH at diagnosis significantly correlated with radiographic progression in patients with seronegative RA after adjusting age, sex, symptom duration, number of active synovitis, and CRP at diagnosis (β*-coefficient* 6.5 ± 1.84; *p* = 0.001 and β-*coefficient* 0.12 ± 0.02; *p* < 0.001, respectively). (4) Conclusions: This study determined that radiographic damage and SvdH at diagnosis were predictive factors in progression of radiographic damage in patients with seronegative rheumatoid arthritis. A large comparative study dedicated to this issue in seronegative RA is required.

## 1. Introduction

Rheumatoid arthritis (RA), which affects 0.4% of the general population, is a chronic autoimmune disease and results in joint damage [1]. RA carries a substantial burden for both patients and society [2]. Based on serological status referring to the presence or absence of rheumatoid factor (RF) and anti-citrullinated protein antibody (ACPA), RA is classified as seropositive or seronegative. RF and ACPA status are important factors for diagnosis, treatment decisions, and prognosis [3,4]. Incidence of seronegative RA is estimated in about 20% of RA patients [1,5]. Seronegative RA has been considered as a less severe clinical entity compared to seropositive RA, with less radiographic damage [6,7,8,9,10]. It has been recommended that patients with seronegative RA should be considered for less intensive treatment than those with seropositive RA, which is also reflected in the 2015 American College of Rheumatology criteria (ACR) [11] and the 2016 European League Against Rheumatism (EULAR) [12] treatment guideline.

However, recent studies demonstrated conflicting results compared to previous reports. Seronegative RA showed higher inflammatory activity at diagnosis than seropositive RA in disease-modifying antirheumatic drug (DMARD)-naïve patients in a cohort study [13]. Lena et al. demonstrated that radiographic progression in seronegative RA was similar to seropositive RA, and treatment response was slower in seronegative patients [14]. Radiographic damage has been reported in 20–42% in patients with seronegative rheumatoid arthritis [5,15]. These results indicate that seronegative RA is not a mild form of the disease and requires intensive treatment similar to that of seropositive RA.

Analytical studies of seronegative RA specifically dedicated to clinical outcomes regarding radiographic progression and related risk factors for that are scarce. Therefore, the aim of this study was to evaluate clinical characteristics at presentation and radiographic outcome in patients with seronegative rheumatoid arthritis. In particular, this study also determined prognostic factors for radiographic progression in patients with seronegative rheumatoid arthritis.

## 2. Materials and Methods

### 2.1. Study Population

In total, 134 adults with RA who reported as seronegative for both RF and ACPA between August 2003 and December 2016 at Jeju National University Hospital were initially included for data collection. Of this, 116 patients who (1) fulfilled the 1987 ACR [16] or 2010 ACR/EULAR [17] classification criteria for RA, (2) were followed-up for more than 2 years; (3) were given plain radiography of joints two or more times, and (4) whose data were available, were ultimately included and retrospectively analyzed based on baseline characteristics, clinical manifestation, and radiographic progression. Patients who showed positivity of high titer for antinuclear antibody, human leukocyte antigen (HLA)-B27 or HLA-B51 with specific extra-articular manifestation fulfilling classification criteria of any other connective tissue diseases during follow-up periods were excluded.

### 2.2. Demographic Variable and Data Collection

Board-certified specialists of rheumatology reviewed medical records, plain radiography, and laboratory findings. Baseline characteristics included demographics, past medical history, smoking, active synovitis at first visit, morning stiffness, symptom duration, erythrocyte sedimentation rate (ESR), C-reactive protein (CRP), joint erosion, and the Sharp van der Heijde (SvdH) score at diagnosis. Symptoms were referred to as joint pain, erythema or swelling.

### 2.3. Outcome Measurement and Radiographic Assessment

The presence of radiographic damage of seronegative RA was evaluated in all patients and was measured blindly by SvdH score during the follow-up periods by two trained specialists of rheumatology [18,19,20]. The Interclass Correlation Coefficients (ICC) of the initial SvdH and the last SvdH score between two reader were 0.95 and 0.96, respectively. Radiographic progression was defined as one or more units change of SvdH score per year following recommendation that one unit of change is the lowest value for minor radiographic change [21].

### 2.4. Statistical Analysis

Statistical analysis was performed using the Statistical Package for Social Science (SPSS) version 21. Descriptive statistics were performed to evaluate the means with standard deviation for continuous variables, and frequencies were calculated for dichotomous data. Univariate and multivariate linear regression analyses were performed to determine the predictive factors for radiographic progression in patients with seronegative RA with or without adjustment of confounding factors. Statistical significance was defined as *p*-value < 0.05.

## 3. Results

### 3.1. Patients Characteristics

Baseline characteristics of seronegative RA with or without radiographic damage are shown in Table 1. A total of 116 patients with seronegative RA were observed and 43 (37.1%) patients demonstrated radiographic damage during follow-up period. Mean age at diagnosis was 48 years and 86 (74.1%) patients were female. Mean follow-up duration of seronegative RA was 5.2 years. Duration of symptoms, which included joint pain, swelling or erythema, at diagnosis was 1.3 years and 109 (94%) of patients showed active synovitis at first visit. Patients with radiographic damage at diagnosis were 14 (12.1%) and mean SvdH score was 6.8 ± 19.4 at diagnosis. Small joints (78.6%) were the most frequently involved in seronegative RA patients with radiographic damage at diagnosis. Follow-up duration is longer in patients with radiographic damage than those without (*p* = 0.02). Fourteen (32.6%) out of 43 patients with radiographic damage during follow-up periods demonstrated joint erosion at diagnosis, and SdvH score of those patients at diagnosis was 36.7 ± 9.8. Other baseline characteristics including sex, age, symptom duration, smoking status, morning stiffness, number of active joints, and acute phase reactant (APR) were not significantly different between the two groups.

### 3.2. Characteristics of Seronegative RA Patients with Radiographic Progression

Characteristics of 43 patients with radiographic progression during follow-up periods are shown in Table 2. The mean SvdH score, which is assessed by plain radiography lastly during follow-up periods, was 47.1 in seronegative RA patients with radiographic progression. The mean change of SvdH score per year in this population was 5.53. In total, 29 (67.4%) of 43 patients with radiographic damage demonstrated joint erosion and joint space narrowing while the rest of the patients (32.6%) showed joint space narrowing only. The radiographic damage that involved small and large joints simultaneously (69.8%) was more frequent compared with those only involved small joints (30.2%). The number of patients with radiographic damage only in hands joints, defined as proximal interphalangeal (PIP), metacarpophalangeal (MCP), 1st interphalangeal (IP) joints of hands and wrist joints, was 4 (9.3%). All patients showed radiographic damage in foot joints, defined as metatarsophalangeal and 1st IP joints of feet, presented simultaneously involvement of other joints. Thirty-nine patients (91.7%) with radiographic damage demonstrated multiple joint involvement.

### 3.3. Predictive Factors of Radiographic Progression in Patients with Seronegative RA

Table 3 demonstrates predictive factors of radiographic progression in seronegative RA. Linear regression analysis was performed to determine predictive factors of radiographic progression, defined as an increase of one or more units of SvdH per year, in seronegative RA patients. Symptoms duration, joint erosion, and SvdH score at diagnosis showed statistical significance in radiographic progression of patients with seronegative RA by univariate linear regression analysis (β*-coefficient* 0.29 ± 0.13; *p* = 0.02, β*-coefficient* 12.61 ± 1.09; *p* < 0.001 and β*-coefficient* 0.21 ± 0.02; *p* < 0.001, respectively). There was no significant association between radiographic progression and other factors such as sex, age, smoking status, morning stiffness, number of active synovitis, and CRP at diagnosis in seronegative RA. Joint erosion and SvdH at diagnosis significantly correlated with radiographic progression in patients with seronegative RA after adjusting age, sex, symptom duration, number of active synovitis, and CRP at diagnosis (β*-coefficient* 6.5 ± 1.84; *p* = 0.001 and β-*coefficient* 0.12 ± 0.02; *p* < 0.001, respectively).

## 4. Discussion

Identifying factors to predict the clinical outcome might be crucial in decision-making for management in patients with RA, because those are closely connected with physical function and quality of life. In this study, 37.1% of 116 patients with seronegative RA showed radiographic damage during follow-up periods. Presence of erosion and SvdH score at diagnosis significantly affect the radiographic progression in seronegative rheumatoid arthritis. Age, sex, smoking status, morning stiffness, and CRP at diagnosis were not associated with radiographic progression in seronegative RA in this data.

Clinical course and outcome of seronegative RA have shown to be conflicting in previous studies. Seronegative RA has been considered as a mild form of the disease, with less radiographic damage [6,7,8,9,10]. In particular, studies of participants classified with RA according to the 1987 ACR criteria indicated that patients with seronegative RA showed less severe radiographic damage compared to those with seropositive RA [6,7,8,9,10,22], and inflammatory activity in seropositive patients was higher than that in seronegative patients [3,7,9]. However, recent studies of RA patients fulfilling the 2010 ACR/EULAR criteria demonstrated that disease activity at the time of diagnosis is higher in seronegative patients because the 2010 criteria puts strong emphasis on serological status [22,23,24,25]. In the ARCTIC (Aiming for Remission in rheumatoid arthritis: a randomized trial examining the benefit of ultrasound in a Clinical TIght Control regimen) trial, included patients with RA classified according to the 2010 ACR/EULAR criteria determined that radiographic damage, disease activity measures, and remission rate were similar between patients with seronegative and seropositive rheumatoid arthritis [14]. Additionally, treatment response was slower in patients with seronegative than seropositive RA, although all patients received similar management. These results suggest that seronegative RA might be a more serious disease than is currently known.

Incidence of radiographic damage in patients with seronegative RA in the current study is 37.1%, which is similar to that of other data [5,15]. Thirty-two percent of patients with radiographic damage presented with joint space narrowing without erosion in our study. Radiographic outcome is one of the most important outcomes in RA patients because it directly related to functional ability and quality of life. However, studies specifically evaluating risk factors for radiographic progression in seronegative RA are very scarce. Subgroup analysis in one prospective study from the Etude et Suivi des POlyarthrites Indifferenciées Récentes (ESPOIR) cohort described that the presence of erosion at baseline affected with radiographic progression, defined as at least 5 van der Heijde modified total Sharp score (mTSS) points at 1 year in patients with early seronegative RA (OR = 5.42 [95% CI 1.14–25.7], *p* = 0.03) [26]. This result is in line with our study in that joint erosion at diagnosis of seronegative RA was a significant predictor of radiographic progression in a long-term perspective. Furthermore, our study confirmed that SvdH score at diagnosis also significantly relates to radiographic progression in seronegative patients. These results imply that seronegative RA is not quite distinct from seropositive RA in terms of structural damage and related risk factors. Patients with erosion and radiographic damage at diagnosis of seronegative RA should be considered for intensive treatment.

Although some factors indicating moderate to high disease activity according to composite measures, such as high swollen joint counts [11,12,27,28] or high acute phase reactant level [11,12,28] have been well known to be associated with poor prognostic outcome in RA patients, studies are rare which analyze specifically the role of these factors for radiographic damage or progression in patients with seronegative rheumatoid arthritis. In several studies of seronegative RA, active joint count or acute phase reactant level at baseline did not show statistical significance for clinical outcome measures. Similarly, the number of active synovitis or acute phase reactant level as a single parameter was not significantly associated with radiographic progression in seronegative patients of current study. A large comparative and prospective study to explore the relationship between these factors and radiographic outcome in seronegative RA is needed.

Several other factors such as medications and novel antibody separately from RF or ACPA, might affect radiographic progression in seronegative RA, although this study could not be analyzed because of study design. Recently published studies reported that the early introduction of the disease-modifying anti-rheumatic drugs (DMARDs) was an independent factor for favorable therapeutic response in seronegative RA [14,29]. Early use of conventional synthetic DMARDs (csDMARDs) was significantly associated with a good or moderate EULAR response at 1 year (odds ratio = 2.41 [95% confidence interval 1.07–5.42], *p* = 0.03) [14]. However, no study described the role of early use of DMARDs as a prognostic factor for radiographic outcome as a long-term aspect in seronegative RA patients. Additional or novel autoantibodies, which are not included in classification of RA nor used in clinical practice, also might explain radiographic damage and progression in seronegative RA [30,31]. One study reported that the presence of antimutated citrullinated vimentin predicted radiographic progression of RA as strongly as for ACPA [6]. Another study demonstrated that 14-3-3eta was significantly higher in seronegative RA patients compared to healthy subjects [32]. Jing et al. reported that anti-carbamylated protein antibody predicts for severe clinical course in ACPA-negative RA patients [33]. We could not analyze the role of those factors above because of retrospective study design. Further large prospective or experimental studies to determine prognostic factors for radiographic outcome in seronegative RA are required.

Limitations of this study included single center and retrospective observational design. Additionally, activity measures such as Disease Activity Score of 28 joints (DAS-28) or functional status measures such as Health Assessment Questionnaire Disability Index (HCQ-DI), which are important possible prognostic factors for radiographic damage in seronegative RA, were not available for analysis due to missing values related to study design. The role of smoking status was not fully analyzed in this study because of missing data related to retrospective study design. Lastly, we could not directly compare manifestation of radiographic damage in seronegative to seropositive RA, apart from predictive factors in radiographic progression. Nevertheless, considering the scarcity of previous data, the strength of the current study is to determine predictive factors of radiographic progression in seronegative RA and to describe different characteristics between seronegative RA patients with or without radiographic damage.

## 5. Conclusions

This study demonstrated a rate of radiographic damage in patients with seronegative RA, which was comparable to those in recent studies. In terms of radiographic progression, this data confirmed that the presence of erosion and SvdH score at diagnosis were predictive factors in patients with seronegative RA. These factors should be considered in making treatment decisions or to predict radiographic outcome in management of seronegative rheumatoid arthritis. A large comparative and prospective study dedicated to this issue in seronegative RA is required.

## Figures and Tables

**Table 1:** Baseline characteristics of seronegative rheumatoid arthritis with or without radiographic damage.

	Total Patients with Seronegative RA (*N* = 116)	Patients with Radiographic Damage (*N* = 43, (37.1%))	Patients without Radiographic Damage (*N* = 73 (62.9%))	*p*
Sex (%)				0.56
Male	30 (25.9)	10 (23.3)	20 (27.6)	
Female	86 (74.1)	33 (76.7)	53 (72.6)	
Age at diagnosis (yrs; mean ± SD)	48 ± 12.2	47.9 ± 11.7	49.1 ± 12.6	0.60
Symptoms duration at diagnosis (yrs; mean ± SD)	1.3 ± 2.1	1.3 ± 2.4	1.2 ± 1.9	0.89
Follow-up duration (yrs; mean ± SD)	5.2 ± 4.0	8.5 ± 4.7	4.5 ± 3.4	0.02
Past history (%)				
Diabetes mellitus	11 (9.6)	2 (4.7)	9 (12.3)	0.28
Hypertension	14 (12.1)	6 (14.0)	8 (11.0)	0.42
Pulmonary tuberculosis	3 (2.6)	2 (4.7)	1 (1.4)	0.55
Hepatitis	5 (4.3)	1 (2.3)	4 (5.5)	0.65
Smoking (%)	20/87 (23.0)	6/34 (17.6)	14/53 (26.4)	0.43
Morning stiffness at diagnosis (%)	92 (79.2)	33 (76.7)	60 (82.2)	0.48
Active synovitis at diagnosis (%)	109 (94.0)	40 (93.0)	69 (94.5)	0.71
Number of joints with active synovitis	7.4 ± 6.8	8.1 ± 7.7	7.9 ± 6.4	0.41
Distribution of active synovitis				0.29
Small joints only	43 (37.1)	11 (25.6)	32 (43.8)	
Large joint only	23 (19.8)	9 (20.9)	14 (19.2)	
Small + Large joints	40 (34.5)	18 (41.9)	22 (30.1)	
ESR at diagnosis (mm/hr)	32.9 ± 28.8	32.5 ± 29.3	32.2 ± 28.2	0.89
CRP at diagnosis (mg/dL)	1.6 ± 2.5	1.6 ± 2.1	1.5 ± 2.6	0.95
Joint erosion at diagnosis (%)	14 (12.1)	14 (32.6)	0 (0)	<0.001
Number of joints with erosion (%)	2.2 ± 1.9	2.2± 1.9		
Distribution of erosion (%)				
Small joints only	11 (78.6)	11 (78.6)		
Hand ^1^ joints only	9 (64.3)	9 (64.3)		
Foot ^2^ joints only	0 (0)	0 (0)		
Hand + foot joints	2 (14.3)	2 (14.3)		
Large ^3^ joint only	1 (7.1)	1 (7.1)		
Elbow	1 (7.1)	1 (7.1)		
Small + large joints	2 (14.3)	2 (14.3)		
Hand and/or foot + large joints	2 (14.3)	2 (14.3)		
Sharp van der Heijde score at diagnosis (mean ± SD)	6.8 ± 19.4	36.7 ± 9.8	0 ± 0	<0.001

Except where indicated otherwise, values are the number (%) of variables. N, number; yrs, years; SD, standard deviation; ESR, erythrocyte sedimentation rate; CRP, C-reactive protein. ^1^ Hand joint included proximal interphalangeal (PIP), metacarpophalangeal (MCP), 1st interphalangeal (IP) joints of hands and wrist joints. ^2^ Foot joint included metatarsophalangeal and 1st IP joints of feet. ^3^ Large joint included elbow, ankle, knee, hip, and shoulder joints.

**Table 2:** Characteristics of seronegative rheumatoid arthritis (RA) patients with radiographic progression during follow-up period (total number of patients = 43).

Characteristics	Number (%)
SvdH score, mean ± SD	47.1 ± 43.7
Change of SvdH score/year, mean ± SD	5.53 ± 8.7
Joint space narrowing only	14 (32.6)
Joint erosion + joint space narrowing	29 (67.4)
Distribution of radiographic damage	
Small joints only	13 (30.2)
Hand ^1^ joints only	4 (9.3)
Foot ^2^ joints only	0 (0)
Hand + foot joints	9 (20.9)
Small + large ^3^ joints	30 (69.8)
Hand and/or foot + large joints	30 (69.8)
Numbers of involvement with radiographic damage	
One joint	4 (9.3)
Multiple joints	39 (91.7)

Except where indicated otherwise, values are the number (%) of variables. SvdH, the Sharp van der Heijde. ^1^ Hand joint included proximal interphalangeal (PIP), metacarpophalangeal (MCP), 1st interphalangeal (IP) joints of hands and wrist joints. ^2^ Foot joint included metatarsophalangeal and 1st IP joints of feet. ^3^ Large joint included elbow, ankle, knee, hip, and shoulder joints.

**Table 3:** Predictive factors of radiographic progression by using univariate and multivariate linear regression analyses in patients with seronegative rheumatoid arthritis.

	Univariable		Multivariable ^1^	
	β ± SE	*p-Value*	β ± SE	*p-Value*
Sex	0.47 ± 1.20	0.69	n.s.	n.s.
Age at diagnosis	0.11 ± 0.04	0.61	n.s.	n.s.
Symptom duration at diagnosis	0.29 ± 0.13	0.02	0.08 ± 0.09	0.42
Smoking at diagnosis	−0.78 ± 1.60	0.63	n.s.	n.s.
Morning stiffness at diagnosis	0.48 ± 0.32	0.71	n.s.	n.s.
Number of active synovitis at diagnosis	0.03 ± 0.08	0.64	n.s.	n.s.
CRP at diagnosis	1.19 ± 0.21	0.34	n.s.	n.s.
Joint erosion at diagnosis	12.61 ± 1.09	<0.001	6.50 ± 1.84	0.001
SvdH score at diagnosis	0.21 ± 0.02	<0.001	0.12 ± 0.02	<0.001

^1^ Adjusted for sex, age, number of active synovitis, symptom duration, CRP, joint erosion, SvdH score at diagnosis. Smoking status was not included multivariate linear regression analysis because of missing values. β, unstandardized regression coefficient; SE, standard error; n.s., nonsignificant; CRP, C-reactive protein; SvdH, the Sharp van der Heijde.

## Data Availability

The datasets generated during and/or analyzed during the current study are available from the corresponding author upon reasonable requests.
